# Persistence of HBsAg‐specific antibodies and immune memory two to three decades after hepatitis B vaccination in adults

**DOI:** 10.1111/jvh.13125

**Published:** 2019-06-02

**Authors:** Pierre Van Damme, Marc Dionne, Geert Leroux‐Roels, Olivier Van Der Meeren, Emmanuel Di Paolo, Bruno Salaun, Pemmaraju Surya Kiran, Nicolas Folschweiller

**Affiliations:** ^1^ Centre for the Evaluation of Vaccination (CEV) Vaccine & Infectious Disease Institute (VAXINFECTIO) University of Antwerp Wilrijk Belgium; ^2^ Centre Hospitalier Universitaire de Québec‐Université Laval Quebec City Quebec Canada; ^3^ Centre for Vaccinology (CEVAC) Ghent University Hospital Ghent Belgium; ^4^ GSK Wavre Belgium; ^5^ GSK Rixensart Belgium; ^6^ GSK Bangalore India

**Keywords:** anamnestic response, HBsAg, hepatitis B vaccine, immune memory, persistence

## Abstract

The duration of protection after hepatitis B vaccination is not exactly known. This phase IV study evaluated antibody persistence and immune memory 20‐30 years after adult immunization with recombinant hepatitis B vaccine (HBsAg vaccine*, Engerix‐B*) in routine clinical practice. Men and women 40‐60 years old, with documented evidence of vaccination with three or four HBsAg vaccine doses 20‐30 years earlier and without subsequent booster, were enrolled and received HBsAg vaccine as challenge dose. HBsAg‐specific antibodies (anti‐HBs) and frequencies of HBsAg‐specific circulating memory B cells and CD4^+^ T cells expressing combinations of activation markers (CD40L, IL2, IFNγ, TNFα) were measured prechallenge, 7 and 30 days postchallenge. Of 101 participants in the according‐to‐protocol cohort for immunogenicity, 90.1% had anti‐HBs concentrations ≥ 10 mIU/mL prechallenge administration; 84.2% and 100% mounted an anamnestic response 7 and 30 days postchallenge, respectively. HBsAg‐specific memory B and CD4^+^ T cells expressing at least two activation markers were low prechallenge and increased markedly postchallenge. These results suggest sustained immune memory and long‐term protection 20‐30 years after a complete primary HBsAg vaccination course during adulthood, in line with current recommendations that a booster is not needed in fully vaccinated immunocompetent adults.

**Abbreviations**
AE: adverse event
anti‐HBc: antibodies against hepatitis B core antigen
anti‐HBs: antibodies against hepatitis B surface antigen
ATP: according‐to‐protocol
CD40L: CD40 ligand
CI: confidence interval
ELISPOT: enzyme‐linked immunosorbent spot
GMC: geometric mean concentration
HBsAg vaccine: recombinant hepatitis B vaccine
HBsAg: hepatitis B surface antigen
HBV: hepatitis B virus
IFNγ: interferon gamma
IL2: interleukin‐2
LLOQ: lower limit of quantification
PBMC: peripheral blood mononuclear cell
SAE: serious adverse event
TNFα: tumour necrosis factor alpha
TVC: total vaccinated cohort
WHO: World Health Organization

## 1 INTRODUCTION

Approximately 292 million individuals (3.9% of the world's population) were living with hepatitis B virus (HBV) infection in 2016.^1^, ^2^ Chronic infection can be asymptomatic for many years and increases the risk of liver cirrhosis and hepatocellular carcinoma. Moreover, chronically infected individuals are a source of transmission of the virus. In 2015, nearly 900 000 people died as a consequence of HBV infection.^1^, ^3^

As the risk of chronic HBV infection is highest when infection is acquired perinatally or during early childhood,^4^ young children are the main target of prevention efforts. The World Health Organization (WHO) recommends hepatitis B vaccination as soon as possible after birth, followed by two or three doses during infancy.^5^ It also recommends vaccinating older age groups at high risk of HBV infection and catch‐up vaccination in previously unvaccinated individuals.^5^ Vaccines containing hepatitis B surface antigen (HBsAg) have been available since the early eighties. Their widespread use in infant immunization programmes has led to a decline in acute and chronic HBV infection and related morbidity and mortality.^1^, ^6^, ^7^, ^8^ The childhood incidence of hepatocellular carcinoma decreased significantly after implementation of universal hepatitis B vaccination in Taiwan and other countries.^9^

While the value of hepatitis B vaccination is evident, the duration of protection after vaccination is not exactly known; based on evidence indicating immune memory and protection against infection up to 30 years after a complete primary series, the WHO does not recommend a booster in adequately vaccinated immunocompetent children and adults.^5^, ^10^, ^11^ Additional studies are needed to fully estimate how long protection lasts; long‐term follow‐up studies after adult vaccination are particularly scarce.

B‐ and T‐cell‐mediated immune memory confers long‐term protection after waning of vaccine‐induced antibodies against HBsAg (anti‐HBs).^12^, ^13^, ^14^ Different methods have been used to assess long‐term HBV protection, including measuring the anamnestic response to a challenge dose, in vitro testing for B‐ and T‐cell activation, or measuring the infection rate in vaccinated populations after extended follow‐up.^14^, ^15^

In this study, we evaluated long‐term protection against HBV infection 20‐30 years after immunization of adults with three or four doses of GSK's recombinant hepatitis B vaccine (HBsAg vaccine, *Engerix‐B*). We quantified the anamnestic response to an HBsAg vaccine challenge dose (primary objective), measured persisting circulating anti‐HBs antibodies, assessed the reactogenicity and safety of the challenge dose (secondary objectives) and evaluated HBsAg‐specific cellular immune memory (tertiary objective). We also checked for previous or ongoing infection.

## 2 METHODS

### 2.1 Study design, participants and vaccines

This phase IV, open‐label, single‐group study was conducted between 11 October 2016 and 1 May 2017 in two centres each in Belgium and Canada. Both countries have a low HBV endemicity (estimated seroprevalence: 0.60%‐0.68% in Belgium and 0.60%‐0.76% in Canada^2^, ^16^). Men and women 40‐60 years old with documented evidence of previous vaccination with three or four consecutive doses of recombinant HBsAg vaccine (*Engerix‐B*, GSK) after 18 years of age were eligible for enrolment if they had received their last dose 4‐12 months after the previous one and 20‐30 years before enrolment, and had not received any hepatitis B booster since. HBsAg vaccination history was verified through personal vaccination cards and vaccination registries and/or by contacting the occupational health department that administered the vaccine. Women of child‐bearing potential could participate if they had practiced adequate contraception for 30 days before vaccination, had a negative pregnancy test on the day of vaccination and agreed to continue adequate contraception during the study. Individuals with immunocompromising conditions, a history of hepatitis B disease or jaundice of unknown origin, drug or alcohol abuse in the last 5 years or acute illness or fever at enrolment were excluded. Inclusion and exclusion criteria are detailed in the Appendix S1. An equal number of participants were planned to be enrolled in two age groups: 40‐50 and 51‐60 years.

Each participant received a single *Engerix‐B* challenge dose (20 μg recombinant HBsAg, 500 μg Al(OH)₃) through intramuscular injection in the deltoid region of the nondominant arm.

The study was conducted according to the principles of Good Clinical Practice, the Declaration of Helsinki and all applicable regulations. The Institutional Review Boards and/or Ethics Committees of the participating centres approved the protocol, informed consent form and other study‐related documents before study start. Each participant provided written informed consent before enrolment. The trial is registered on ClinicalTrials.gov (NCT02901951). A protocol summary is available at https://www.gsk-clinicalstudyregister.com/search/?study_ids=116811.

### 2.2 Immunogenicity assessment

Blood samples to assess immunogenicity were collected prechallenge and 7 and 30 days postchallenge. Approximately 5 mL of blood per participant was collected at each visit to measure humoural immune responses. After centrifugation, serum samples were stored at −20°C until shipment to the designated laboratories. Anti‐HBs antibodies were measured at GSK, Rixensart/Wavre, Belgium, using a chemiluminescence assay (*ADVIA Centaur* anti‐HBs2, Siemens Healthcare) with a cut‐off of 6.2 mIU/mL defining seropositivity. Seroprotection has been defined as an antibody concentration of ≥ 10 mIU/mL after a complete vaccination course.^17^, ^18^, ^19^ An anamnestic response was defined as a ≥ 4‐fold rise in anti‐HBs concentrations 7 or 30 days postchallenge in previously seropositive individuals or an anti‐HBs concentration ≥ 10 mIU/mL 7 or 30 days postchallenge in previously seronegative individuals (ie, with undetectable anti‐HBs < 6.2 mIU/mL).

Antibodies against hepatitis B core antigen (anti‐HBc) were measured at the prechallenge timepoint to assess previous or ongoing infection, using a qualitative chemiluminescence assay (*Immulite*, Siemens Healthcare) at the Center for Vaccinology, Ghent, Belgium.

Approximately 27 mL of blood per participant was collected in heparinized tubes at each visit to measure the HBsAg‐specific cell‐mediated immune response. Whole blood samples were stored and shipped at room temperature to certified peripheral blood mononuclear cell (PBMC)‐processing laboratories in Belgium and Canada for PBMC separation to be performed within 24 hours of blood collection. Frequencies of memory B cells secreting anti‐HBs were measured using a B‐cell enzyme‐linked immunosorbent spot (ELISPOT) assay at GSK, Rixensart/Wavre, Belgium, as previously described.^20^, ^21^ Frequencies of HBsAg‐specific CD4^+^ T cells expressing various combinations of activation markers (CD40 ligand [CD40L], interleukin‐2 [IL‐2], interferon gamma [IFNγ] and tumour necrosis factor alpha [TNFα]) were measured by intracellular cytokine staining and cytokine flow cytometry on PBMCs after stimulation with HBsAg at GSK, Rixensart/Wavre, Belgium, as previously described.^22^ The lower limit of quantification (LLOQ) was 354 HBsAg‐specific CD4^+^ T cells per million CD4^+^ T cells. Cell‐mediated immune responses were not analysed on samples from one centre in Canada because of a high cell death due to technical issues before sample processing.

### 2.3 Reactogenicity and safety assessment

The reactogenicity and safety of the HBsAg vaccine challenge dose were assessed in terms of solicited symptoms, unsolicited adverse events (AEs) and serious AEs (SAEs). Each participant received a diary card to record solicited local (injection site) and general symptoms occurring within 4 days postchallenge and unsolicited AEs occurring within 31 days postchallenge. The intensity of all AEs was graded on a scale from 1 (mild) to 3 (severe).

### 2.4 Statistical analyses

All objectives were descriptive. Immunogenicity was assessed on the according‐to‐protocol (ATP) cohort for immunogenicity, including all eligible participants complying with the protocol‐defined procedures and intervals (5‐12 days between the pre‐ and 7 days postchallenge timepoints and 21‐48 days between the pre‐ and 30 days postchallenge timepoints), who received the HBsAg vaccine challenge dose, and for whom immunogenicity results prechallenge and 30 days postchallenge were available. The percentage of participants with anti‐HBs concentrations ≥6.2 mIU/mL, ≥10 mIU/mL and ≥ 100 mIU/mL and the percentage who mounted an anamnestic response to the challenge dose were calculated with exact 95% confidence intervals (CIs). Antibody geometric mean concentrations (GMCs), with 95% CIs, were calculated by taking the antilog of the mean of the log10 concentration transformations, with an arbitrary value of half the assay cut‐off given for concentrations below the 6.2 mIU/mL cut‐off. Analyses were also performed by prechallenge antibody concentration and age.

The frequencies of HBsAg‐specific memory B cells and CD4^+^ T cells expressing various combinations of CD40L, IL2, IFNγ and/or TNFα were evaluated for the ATP cohort for immunogenicity and separately for the two age groups. For geometric mean frequency calculations for memory B cells, values of 0 were given an arbitrary value of 1. HBsAg‐specific cellular immunity was originally defined as a secondary objective but was downgraded to tertiary at the time of analysis because data were exploratory and no correlate of protection has been established for cellular immunity. The correlation of HBsAg‐specific memory B and CD4^+^ T cells with postchallenge anti‐HBs concentrations and with the amplitude of the anamnestic response (ie, fold change post‐ vs prechallenge) was analysed by Spearman's rank correlation coefficient.

Reactogenicity and safety were assessed on the total vaccinated cohort (TVC), including all participants who received the HBsAg vaccine challenge dose. Percentages of participants reporting each AE were calculated with exact 95% CIs.

The sample size was estimated based on an acceptable precision of the anamnestic response in terms of exact 95% CIs. Assuming a dropout rate of 20%, 100 participants were planned to be enrolled to obtain 80 evaluable participants.

## 3 RESULTS

### 3.1 Study participants

A total of 103 adults were enrolled, received an HBsAg vaccine challenge dose and completed the study. All but two participants were included in the ATP cohort for immunogenicity: one was eliminated because of a positive anti‐HBc test result and one because of incoherent anti‐HBs results. In the TVC, the mean age was 48.6 years; 60 participants were 40‐50 years old and 43 were 51‐60 years old (Table 1). Most participants (84.5%) were female. A total of 49 participants had received a prior three‐dose HBsAg vaccination schedule, with a mean interval between the last dose and the challenge dose of 22.9 years (range: 19.0‐27.0 years). A total of 54 participants had received four prior doses, with a mean interval between the last dose and the challenge dose of 24.9 years (range: 20.0‐30.0 years). The mean, minimum and maximum duration between the last dose and the challenge dose was in the same range for the two age groups (Table 1).

**Table 1:** Baseline characteristics and HBsAg vaccination history of study participants by age group and overall (total vaccinated cohort)

Characteristic^a^	40‐50 years N = 60	51‐60 years N = 43	Total N = 103
Mean age ± SD at challenge dose, years	44.3 ± 3.1	54.7 ± 2.6	48.6 ± 5.9
Sex, n (%)			
Female	54 (90.0)	33 (76.7)	87 (84.5)
Male	6 (10.0)	10 (23.3)	16 (15.5)
Geographic ancestry, n (%)			
White—Caucasian/European heritage	60 (100)	43 (100)	103 (100)
BMI ± SD, kg/m^2^	25.7 ± 4.4	24.6 ± 3.0	25.2 ± 3.9
Age at HBsAg vaccine dose 1, years			
Mean	20.1	27.7	23.2
Median (min‐max)	20.0 (17.0‐26.0)	28.0 (20.0‐35.0)	21.0 (17.0‐35.0)
Prior three‐dose HBsAg vaccine regimen			
Number of participants, n	36	13	49
Time from last dose to challenge, years			
Mean	22.8	23.4	22.9
Median (min‐max)	22.5 (19.0^b^27.0)	23.0 (20.0‐26.0)	23.0 (19.0^b^27.0)
Prior four‐dose HBsAg vaccine regimen			
Number of participants, n^c^	24	30^c^	54^c^
Time from last dose to challenge, years			
Mean	23.2	26.3	24.9
Median (min‐max)	23.0 (20.0‐28.0)	27.0 (21.0‐30.0)	25.0 (20.0‐30.0)

Abbreviations: BMI, body mass index; HBsAg, hepatitis B surface antigen; N, total number of participants; SD, standard deviation; n (%), number (percentage) of participants per category.

a Age categories 41‐50 and 51‐60 years are defined based on the age at the challenge dose.

b For one participant, the time between the last dose and the challenge dose was 10 days short of the required 20 years but the participant was still included in the analyses.

c For one participant in the 51‐60 years age group receiving a four‐dose HBsAg vaccination schedule, data on the time between the last dose and the challenge dose were missing; the time was calculated for the remaining 29 participants (or 53 in the age groups combined).

### 3.2 Immunogenicity

#### 3.2.1 Humoral immunity

Twenty to thirty years after three‐ or four‐dose vaccination with HBsAg vaccine, 90.1% of participants had an anti‐HBs concentration ≥ 10 mIU/mL and 61.4% had a concentration ≥ 100 mIU/mL (Table 2).

**Table 2:** Percentage of participants with anti‐HBs concentrations ≥ 6.2 mIU/mL, ≥10 mIU/mL and ≥ 100 mIU/mL, anamnestic response and anti‐HBs GMCs, overall and by prechallenge serostatus (ATP cohort for immunogenicity)

Timepoint	Prechallenge serostatus	N	% ≥6.2 mIU/mL (95% CI)	% ≥10 mIU/mL (95% CI)	% ≥100 mIU/mL (95% CI)	Anamnestic response, % (95% CI)	Antibody GMC, mIU/mL (95% CI)
Prechallenge	All	101	94.1 (87.5‐97.8)	90.1 (82.5‐95.1)	61.4 (51.2‐70.9)	Not applicable	184.6 (121.5‐280.3)
7 days postchallenge	All	101	98.0 (93.0‐99.8)	97.0 (91.6‐99.4)	92.1 (85.0‐96.5)	84.2 (75.6‐90.7)	3840.0 (2330.0‐6328.6)
	<10 mIU/mL	10	80.0 (44.4‐97.5)	70.0 (34.8‐93.3)	50.0 (18.7‐81.3)	70.0 (34.8‐93.3)	82.9 (13.5‐510.4)
	≥10 mIU/mL	91	100.0 (96.0‐100.0)	100.0 (96.0‐100.0)	96.7 (90.7‐99.3)	85.7 (76.8‐92.2)	5852.9 (3734.1‐9173.8)
30 days postchallenge	All	101	100.0 (96.4‐100.0)	100.0 (96.4‐100.0)	98.0 (93.0‐99.8)	100.0 (96.4‐100.0)	48999.1 (33572.7‐71513.7)
	<10 mIU/mL	10	100.0 (69.2‐100.0)	100.0 (69.2‐100.0)	80.0 (44.4‐97.5)	100.0 (69.2‐100.0)	1545.9 (349.5‐6838.5)
	≥10 mIU/mL	91	100.0 (96.0‐100.0)	100.0 (96.0‐100.0)	100.0 (96.0‐100.0)	100.0 (96.0‐100.0)	71636.2 (52708.8‐97360.4)

Abbreviations: Anti‐HBs, antibodies against hepatitis B surface antigen; ATP, according‐to‐protocol; CI, confidence interval; GMC, geometric mean concentration; N, number of participants with available results; %, percentage of participants with concentration equal to or above specified value, or percentage mounting an anamnestic response, defined as a postchallenge antibody concentration ≥ 10 mIU/mL for initially seronegative participants and a postchallenge antibody concentration at least four times the prechallenge antibody concentration for initially seropositive participants (cut‐off for seropositivity: 6.2 mIU/mL).

**Figure 1:**
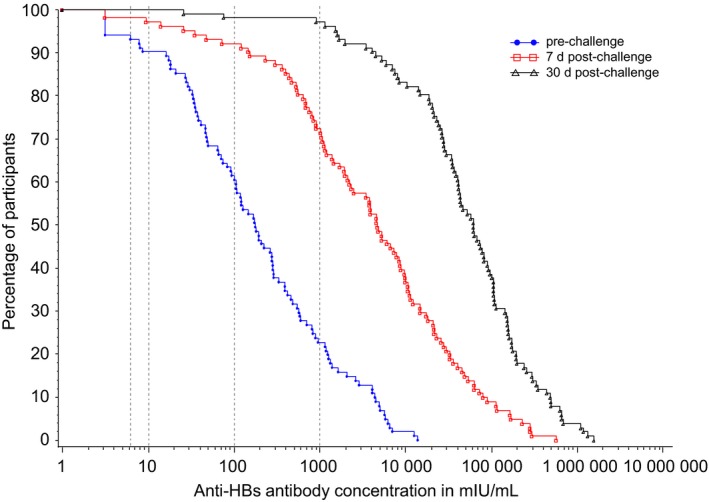
Reverse cumulative distribution curves of anti‐HBs concentrations before, 7 and 30 days after the challenge dose (ATP cohort for immunogenicity). Anti‐HBs, antibodies against hepatitis B surface antigen; ATP, according‐to‐protocol

**Figure 2:**
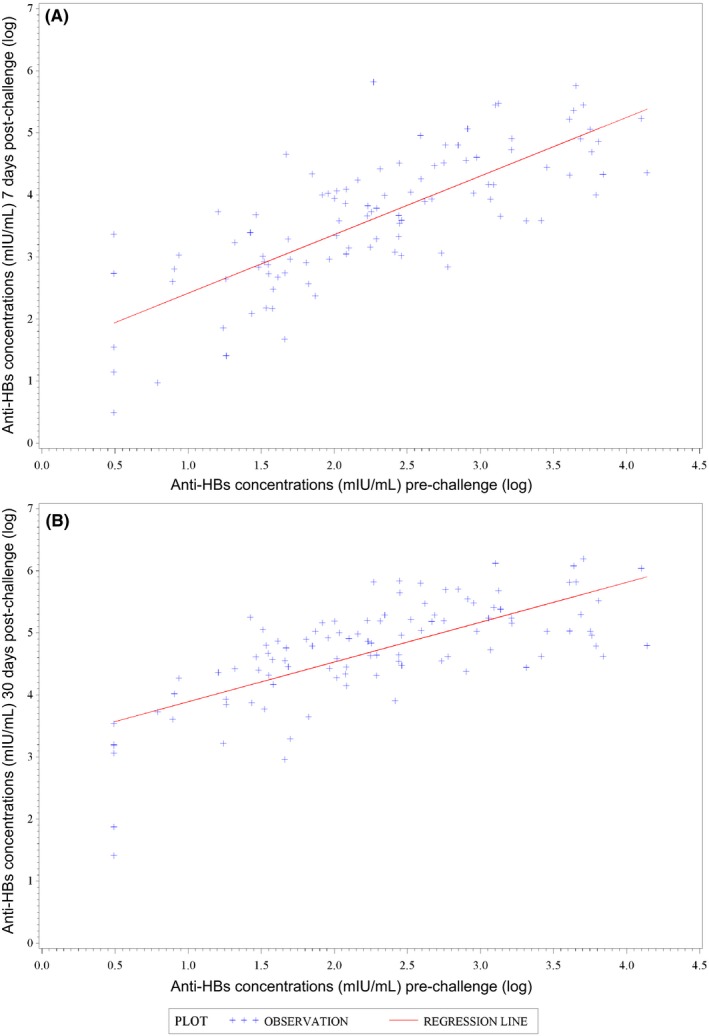
Anti‐HBs concentrations 7 days (A) and 30 days (B) postchallenge as a function of prechallenge concentrations, with regression line (ATP cohort for immunogenicity). Anti‐HBs, antibodies against hepatitis B surface antigen; ATP, according‐to‐protocol

Seven days postchallenge, overall 84.2% of participants mounted an anamnestic response, that is, 70.0% of those who had anti‐HBs levels < 10 mIU/mL prechallenge and 85.7% of those who had anti‐HBs levels ≥ 10 mIU/mL. Thirty days postchallenge, all participants had an anamnestic response regardless of their prechallenge serostatus (Table 2). As a result, 7 days postchallenge, 97.0% of participants had an anti‐HBs concentration ≥ 10 mIU/mL and 92.1% had an antibody concentration ≥ 100 mIU/mL. Thirty days postchallenge, these percentages increased to 100% and 98.0%, respectively (Table 2). Among the 10 participants who had anti‐HBs concentrations < 10 mIU/mL prechallenge, all had reached concentrations ≥ 10 mIU/mL 30 days postchallenge (Table 2). Anti‐HBs concentrations increased from prechallenge to 7 and 30 days postchallenge (Table 2 and Figure 1). Postchallenge anti‐HBs concentrations increased with rising prechallenge concentrations (Figure 2), while fold changes between the pre‐ and 30 days postchallenge timepoints tended to decrease with rising prechallenge concentrations > 100 mIU/mL (Figure S1).

Results were similar for both age groups (Table S1).

#### 3.2.2 Cell‐mediated immunity

**Figure 3:**
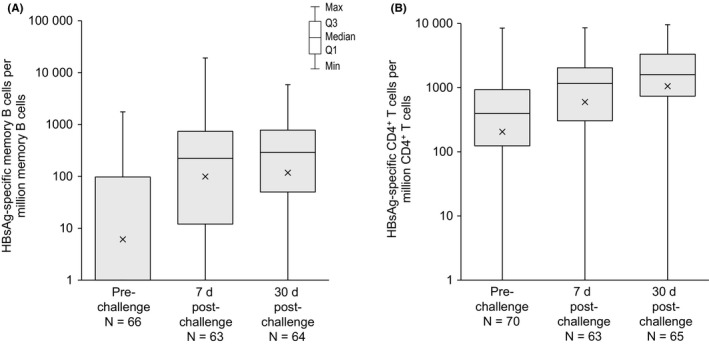
Frequencies of HBsAg‐specific IgG‐producing memory B cells per million of IgG‐producing memory B cells (A) and of HBsAg‐specific CD4^+^ T cells expressing a combination of at least two activation markers (among CD40L, IL2, IFNγ and TNFα) per million of CD4^+^ T cells (B) (ATP cohort for immunogenicity). HBsAg, hepatitis B surface antigen; IgG, immunoglobulin G; CD40L, CD40 ligand; IL2, interleukin 2; IFNγ, interferon gamma; TNFα, tumour necrosis factor alpha; ATP, according‐to‐protocol; N, number of participants with available results; Q1‐Q3, interquartile range. The x symbols in the boxes depict geometric mean frequencies

The frequency of circulating HBsAg‐specific memory B cells was low prechallenge and increased substantially postchallenge (16‐ and 19‐fold increase in geometric mean frequencies from pre‐ to 7 and 30 days postchallenge, respectively) (Figure 3A). HBsAg‐specific CD4^+^ T cells expressing a combination of at least two activation markers (among CD40L, IL2, IFNγ and TNFα) were detected at low frequencies 20‐30 years after primary vaccination, the geometric mean frequency being lower than the assay's LLOQ. The frequency increased postchallenge (3‐ and 5‐fold increase in geometric mean frequencies by 7 and 30 days postchallenge, respectively) (Figure 3B).

No consistent differences were observed between the age groups (Figure S2).

#### 3.2.3 Relation between cell‐mediated and humoural immunity

The frequency of HBsAg‐specific memory B cells postchallenge showed a positive correlation with anti‐HBs concentrations at the respective postchallenge timepoints (Spearman's correlation coefficients of 0.76 and 0.54 with 95% CIs excluding 0 at 7 and 30 days postchallenge, respectively; Table S2). Correlation coefficients between the prechallenge frequency of memory B cells and postchallenge anti‐HBs concentrations were 0.34 and 0.26 (95% CIs excluding 0). Correlation coefficients between the frequency of HBsAg‐specific CD4^+^ T cells expressing at least two cytokine markers pre‐ or postchallenge and anti‐HBs concentrations postchallenge ranged between 0.14 and 0.40. Correlation coefficients between HBsAg‐specific memory B or CD4^+^ T cells and the amplitude of the anamnestic response ranged between ‐0.22 and 0.41 (Table S2).

#### 3.2.4 Previous or ongoing infection

One participant tested positive for anti‐HBc antibodies prechallenge dose. However, an anti‐HBc retest and an HBsAg test were both negative, suggesting that the first anti‐HBc test was a false positive. The participant was nevertheless removed from the ATP cohort for immunogenicity.

### 3.3 Reactogenicity and safety

The challenge dose was well tolerated; no grade 3 symptoms or fever were reported (Table S3). Forty‐one participants (39.8%, 95% CI: 30.3‐49.9) reported at least one unsolicited AE. No SAEs were reported.

## 4 DISCUSSION

This phase IV study in two countries with a low HBV endemicity showed that 90.1% of individuals who had received a complete three‐ or four‐dose primary HBsAg vaccination series during adulthood (in routine daily practice) had anti‐HBs levels ≥ 10 mIU/mL 20‐30 years later. All participants mounted an anamnestic response within 30 days of receiving an HBsAg vaccine challenge dose during the study, including the 9.9% of participants who had anti‐HBs concentrations < 10 mIU/mL prechallenge. Both HBsAg‐specific memory B and CD4^+^ T cells increased markedly postchallenge. These results indicate that the immune response can still be boosted 20‐30 years after vaccination and suggest sustained immune memory and long‐term protection 20‐30 years after a complete primary HBsAg vaccination course in immunocompetent adults.

HBsAg seroprevalence is low in Belgium and Canada, with estimates around 0.6% to 0.8%.^2^, ^16^ The likelihood of HBV exposure resulting in natural boosting is therefore low. In addition, individuals who had received a hepatitis B booster dose after the initial vaccination course were excluded from the study. The high percentage of participants with anti‐HBs concentrations ≥ 10 mIU/mL before challenge administration in our study was therefore most likely due to persistent antibodies induced by the primary HBsAg vaccination series 20‐30 years earlier.

A similarly high anti‐HBs persistence (≥92.0% with antibody levels ≥ 10 mIU/mL) was noted 20 years after three‐dose vaccination with a combined hepatitis A and B vaccine in adults in two long‐term follow‐up studies in Belgium.^23^ Persistence levels in these studies and the current one were higher than in other studies assessing long‐term protection after hepatitis B vaccination during adulthood. A study following a prospective cohort of Alaska Native adults and children immunized with three doses of plasma‐derived hepatitis B vaccine showed that 60% and 50% of participants had anti‐HBs levels ≥ 10 mIU/mL 22 and 30 years after the primary series, respectively.^24^, ^25^ In a study in the United States in which healthcare workers were enrolled 10‐31 years after they received a three‐dose series of either plasma‐derived or recombinant hepatitis B vaccine between the ages of 18 and 60 years, 77% of participants had anti‐HBs levels > 12 mIU/mL.^26^ Among participants enrolled > 20 years after vaccination, this percentage was similar (76%). The lower anti‐HBs persistence in these studies may be due to the different vaccines used, a less effective schedule or underlying genetic differences between the study populations.

Studies evaluating long‐term protection after recombinant HBsAg vaccination during infancy or childhood have shown lower percentages of individuals with persistent anti‐HBs levels ≥ 10 mIU/mL compared to our study. Three studies in Thailand evaluating anti‐HBs persistence 20 years after different infant vaccination schedules with GSK's recombinant HBsAg vaccine showed anti‐HBs levels ≥ 10 mIU/mL in 44.0%‐64.0% of participants.^27^, ^28^, ^29^ In a study evaluating persistence and immune memory after three‐dose vaccination with GSK's recombinant HBsAg vaccine in preadolescents in Quebec, Canada, 76.7% of participants had anti‐HBs levels ≥ 10 mIU/mL 15 years postvaccination.^30^

In all of these studies, as in our current study, the majority of participants mounted an anamnestic response to a hepatitis B challenge, including those whose anti‐HBs levels had declined below 10 mIU/mL, indicating that protection lasts even after waning of anti‐HBs antibodies.^24^, ^25^, ^26^, ^27^, ^28^, ^29^, ^30^

Different definitions and timepoints have been used to measure the anamnestic response to a challenge dose. An HBsAg vaccine challenge has been shown to induce a typical anamnestic response with a steep increase starting between 3 and 7 days postchallenge, peaking around 14 days and plateauing between 14 days and 1 month.^15^ This is consistent with results from our study which show an initial increase in anti‐HBs concentrations 7 days postchallenge (with an 84.2% anamnestic response rate) and a further increase 30 days postchallenge.

In our age‐stratified analyses, the percentage of participants with anti‐HBs levels ≥ 10 mIU/mL and antibody GMCs 20‐30 years after HBsAg vaccination and the anamnestic response to the challenge were in similar ranges for the two age groups (40‐50 and 51‐60 years). This may not be surprising since the maximum age at the first primary dose in the older age group in our study was 35 years and a decrease in the anti‐HBs response to vaccination has been primarily shown for individuals older than 40 years.^31^, ^32^ In addition, anti‐HBs levels in the older age group in our study may have been impacted by the higher proportion of participants in this age group receiving a four‐dose HBsAg vaccination course. Of note, the time between the last dose of the primary course and the challenge dose was similar for both age groups.

Long‐term protection after hepatitis B vaccination can also be assessed by measuring in vitro activation of HBsAg‐specific B and T cells.^14^ The strong anamnestic response observed in our study is indicative of sustained B‐cell memory; long‐lived memory B cells induced after initial antigen exposure remain capable of rapid proliferation, differentiation and secretion of specific antibodies upon subsequent antigen exposure.^33^, ^34^ Consistently, despite low circulating memory B‐cell levels at the prechallenge timepoint, we observed an increase in circulating memory B cells capable of producing anti‐HBs antibodies as soon as 7 days postchallenge and a positive correlation between the frequency of HBsAg‐specific memory B cells and anti‐HBs concentrations at the postchallenge timepoints. The frequency of CD4^+^ T cells producing different cytokines after in vitro stimulation with HBsAg was also measured. Despite low frequencies of HBsAg‐specific CD4^+^ T cells in venous blood 20‐30 years after primary vaccination, the observed increase as early as 7 days after the HBsAg vaccine challenge indicates a memory recall. Previous studies have assessed long‐term persistence of memory B and CD4^+^ T cells and the cellular immune response to a challenge up to 32 years after priming in adults, infants or children.^15^, ^35^, ^36^, ^37^, ^38^, ^39^, ^40^ However, there was substantial heterogeneity in the assays and timepoints used for assessment; therefore, it is hard to compare results.

Long‐term protection can also be assessed by measuring the rate of breakthrough HBV infection in a vaccinated population.^14^ Anti‐HBc antibodies develop after acute HBV infection and generally persist for life. The presence of anti‐HBc antibodies therefore indicates HBV infection at one point in time.^41^ In our study, no information on the prevaccination anti‐HBc status was available; therefore, a positive anti‐HBc test would not be an indication of vaccine failure as infection might have occurred before HBsAg vaccination. Moreover, the positive anti‐HBc test in one participant in our study was probably false positive.

Our study was limited by a sample size of 101 participants. Moreover, all objectives were descriptive; therefore, results should be interpreted with caution. More than 80% of participants were women (because most participants were healthcare workers and women are predominant in this sector), which may limit the generalizability of our results. It has been shown that higher immune responses to hepatitis B primary vaccination are induced in women than men.^32^ As peak levels after priming correlate with long‐term persistence, the high proportion of female participants may have contributed to the high persistence observed in this study. However, no immunological data were available on the original response after the primary course. Correlating antibody persistence or anamnestic responses to the postprimary anti‐HBs response was therefore impossible. This reflects real‐world daily practice where occupational health physicians are often confronted with vaccinees for whom information on the primary immunological response to vaccination is lacking. The small sample size precluded us from obtaining meaningful results from subgroup analyses that would evaluate the influence of sex, body mass index, the number of primary doses (three or four) or time since primary vaccination on anti‐HBs persistence or anamnestic response. Given that 90.1% of participants had anti‐HBs levels ≥ 10 mIU/mL prechallenge and all participants mounted an anamnestic response, we can infer that a full vaccination course conferred long‐term protection regardless of these factors.

**Figure 4:**
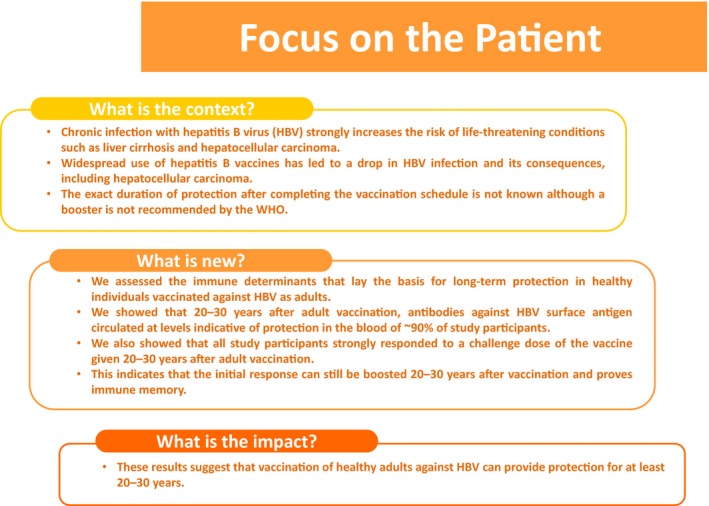
Summary of the research, its clinical relevance and impact on public health

In summary, this study shows anti‐HBs immune memory and antibody persistence 20‐30 years after three or four doses of recombinant HBsAg vaccine administered in adulthood. These results support the current recommendations that a booster dose is not needed in healthy, fully vaccinated, immunocompetent adults.^5^, ^12^, ^42^ Figure 4 summarizes the research, its clinical relevance and impact on public health.

## CONFLICT OF INTEREST

OVDM, EDP, BS, PS and NF are employees of the GSK group of companies; OVDM, EDP, BS and NF own shares in the GSK group of companies; and the institutions of PVD, MD and GLR received grants from the GSK group of companies to conduct the study.

## DATA SHARING

Anonymized individual participant data and study documents can be requested for further research from http://www.clinicalstudydatarequest.com

## AUTHOR CONTRIBUTIONS

OVDM, NF, PVD and GLR were involved in the conception and design of the study. OVDM, EDP, NF, BS, PVD, GLR and MD performed the study. PS, EDP, NF, BS, PVD, GLR and MD were involved in the analysis and interpretation of the data. All authors have critically reviewed and approved the manuscript.

## TRADEMARKS

Engerix‐B is a trademark of the GSK group of companies.
