# Supplementary figures and images for: Persistence of HBsAg‐specific antibodies and immune memory two to three decades after hepatitis B vaccination in adults
Source: J Viral Hepat. 2019 Jun 2;26(9):1066–75. doi: 10.1111/jvh.13125 (PMC6852111; doi:10.1111/jvh.13125)

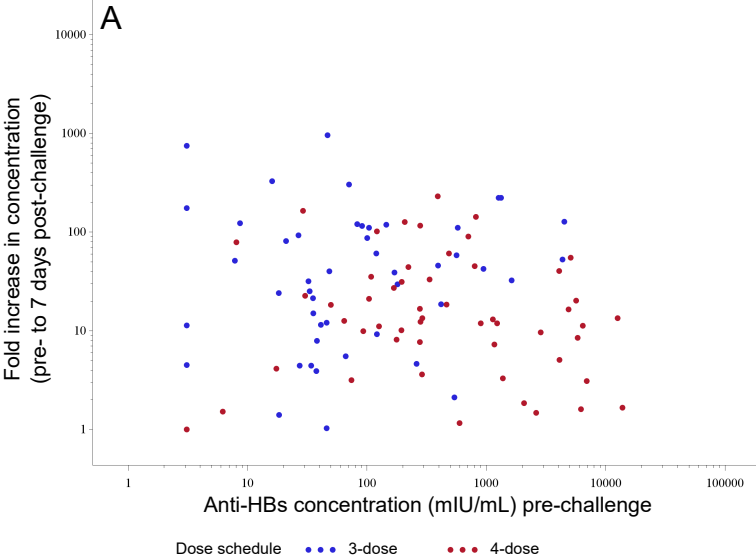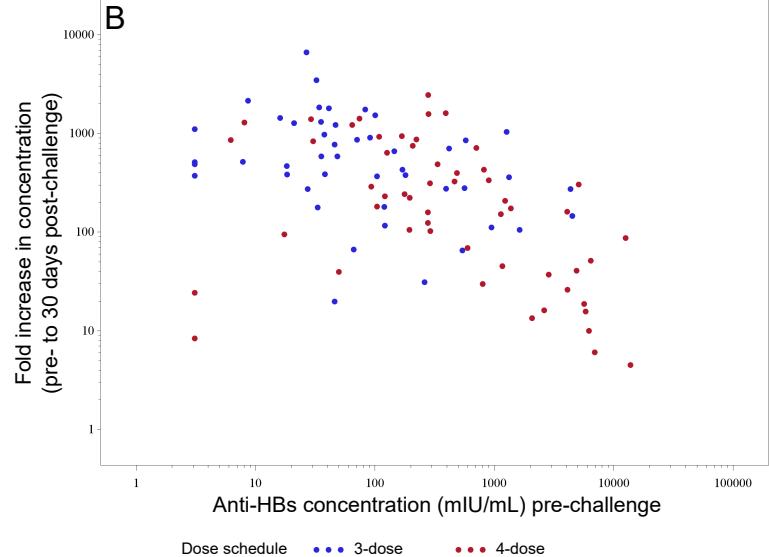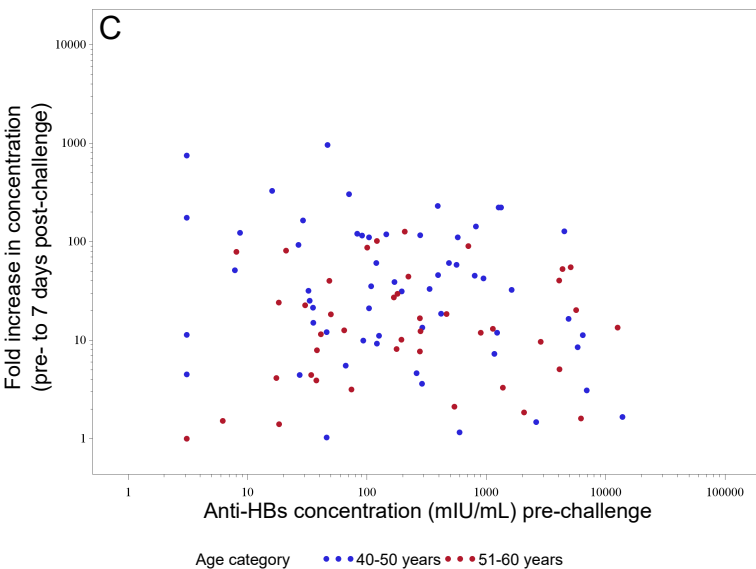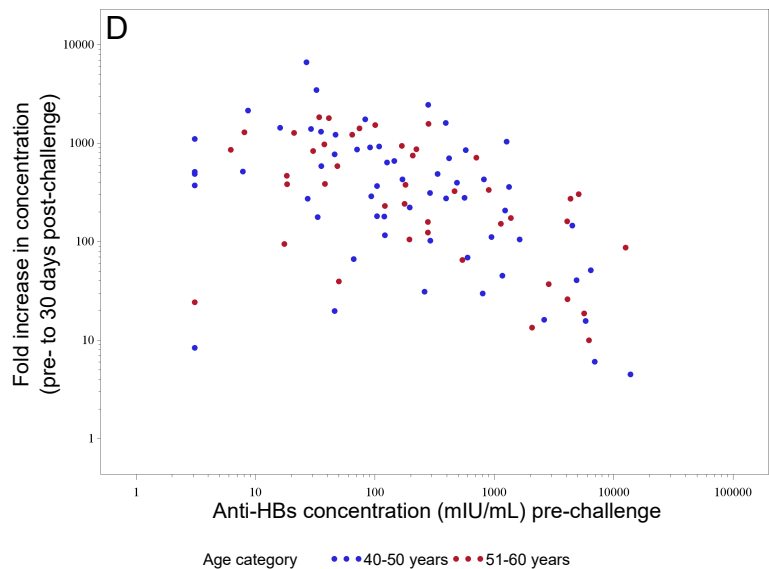

Supplement: Supplementary file 1 [file JVH-26-1066-s001.pdf]

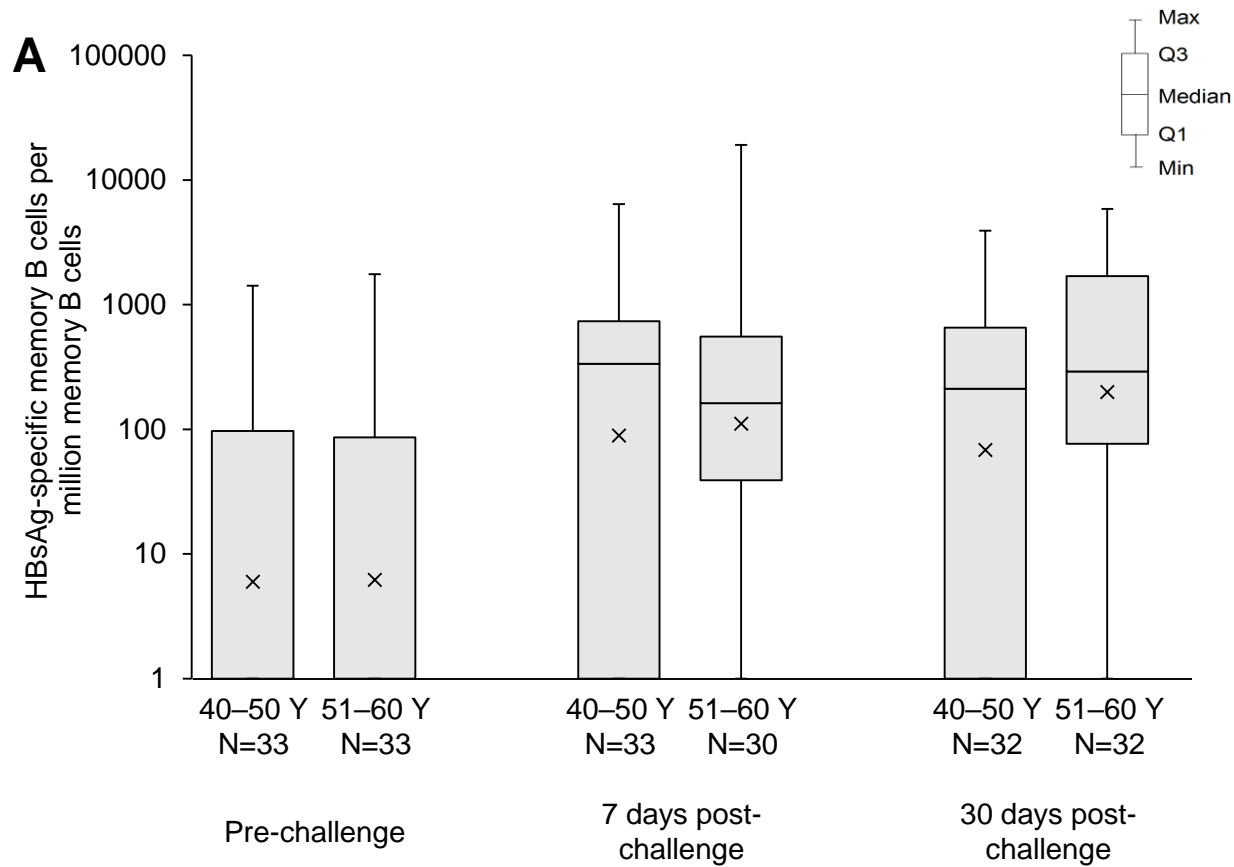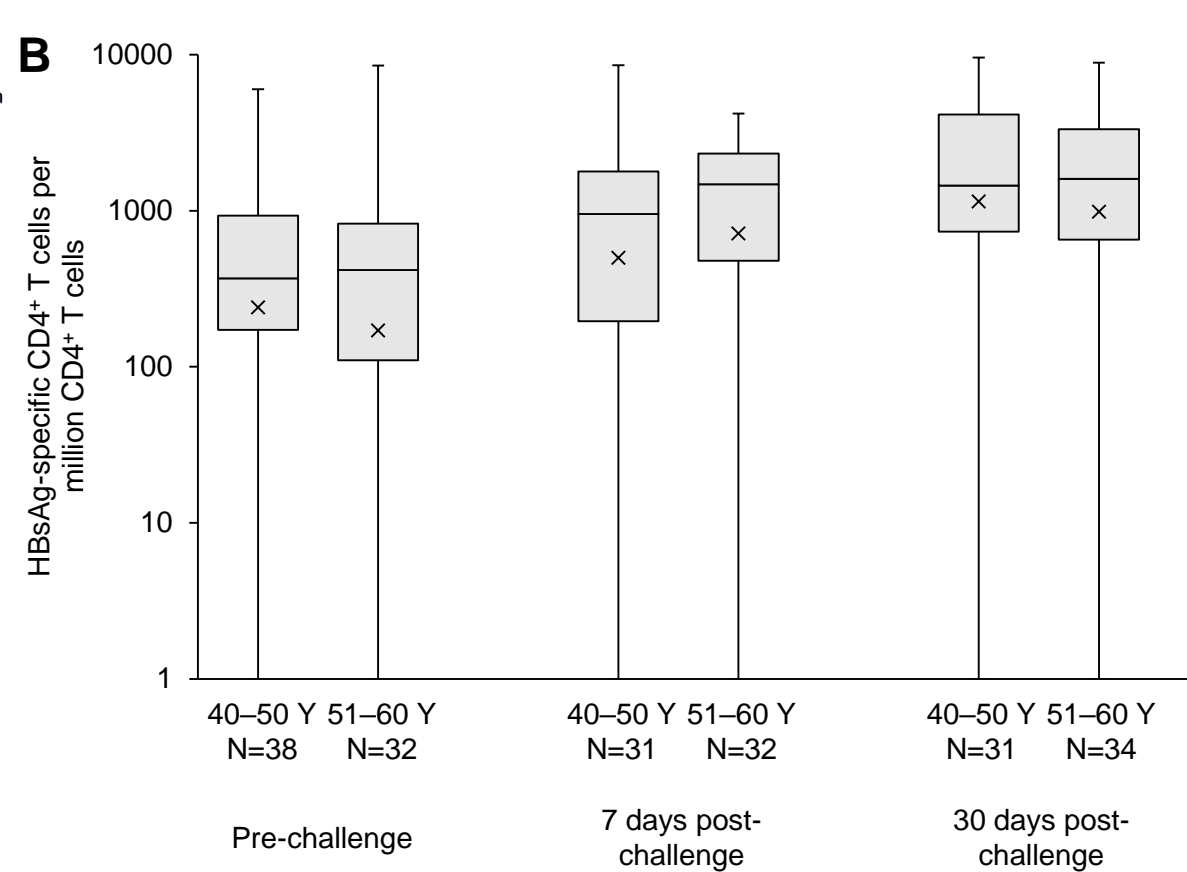

Supplement: Supplementary file 2 [file JVH-26-1066-s002.pdf]
