# Supplementary material for: Persistence of HBsAg‐specific antibodies and immune memory two to three decades after hepatitis B vaccination in adults
Source: J Viral Hepat. 2019 Jun 2;26(9):1066–75. doi: 10.1111/jvh.13125 (PMC6852111; doi:10.1111/jvh.13125)
Supplement: Supplementary file 3 [file JVH-26-1066-s003.docx]

**Supporting information**

**Supplementary methods**

**Inclusion and exclusion criteria**

All subjects had to satisfy all the following criteria at study entry:

- Subjects who, in the opinion of the investigator, could and would comply with the requirements of the protocol (e.g., completion of the diary cards, return for follow-up visits).
- A male or female between, and including, 40 and 60 years of age (from and including the 40^th^ birthday up to, but excluding, the 61^st^ birthday) at the time of the vaccination.
- Written informed consent obtained from the subject.
- Documented evidence of previous vaccination with three or four consecutive doses of *Engerix-B* administered in adulthood (i.e., at least 18 years of age) with
  - the last dose received 4 to 12 months after the previous one,
  - no subsequent booster dose ever received later, and
  - the last dose received 20 to 30 years before enrolment.
- Female subjects of non-childbearing potential could be enrolled in the study.
  - Non-childbearing potential was defined as pre-menarche, hysterectomy, ovariectomy or post-menopause.
- Female subjects of childbearing potential could be enrolled in the study, if the subject:
  - had practiced adequate contraception for 30 days prior to vaccination, and
  - had a negative pregnancy test on the day of vaccination, and
  - had agreed to continue adequate contraception during the entire treatment period and for one month after vaccination.

The following criteria were to be checked at the time of study entry. If any exclusion criterion applied, the subject was not included in the study:

- Use of any investigational or non-registered product (drug or vaccine) other than the study vaccine during the period starting 30 days before the dose of study vaccine, or planned use during the study period.
- Chronic administration (defined as more than 14 days in total) of immunosuppressants or other immune-modifying drugs within six months prior to the vaccine dose. For corticosteroids, this would mean prednisone ≥20 mg/day, or equivalent. Inhaled and topical steroids were allowed.
- Administration of long-acting immune-modifying drugs at any time during the study period (e.g., infliximab).
- Previous hepatitis B booster vaccination since completion of the primary vaccination series with three or four doses of *Engerix-B*.
- Planned administration of a vaccine not foreseen by the study protocol within 30 days preceding the dose of study vaccine, or planned administration during the study period, with the exception of seasonal influenza vaccine.
- Any medical condition that in the judgment of the investigator placed the subject at undue risk by participating in the study.
- Concurrently participating in another clinical study, at any time during the study period, in which the subject had been or was exposed to an investigational or a non-investigational vaccine/product (pharmaceutical product or device).
- History of hepatitis B disease or episode of jaundice with unknown etiology.
- History of any reaction or hypersensitivity likely to be exacerbated by any component of the vaccine.
- Any confirmed or suspected immunosuppressive or immunodeficient condition, based on medical history and physical examination (no laboratory testing required).
- Major congenital defects or serious chronic illness (including insulin-dependent diabetes).
- Acute disease and/or fever at the time of enrolment.
  - Fever was defined as temperature ≥ 37.5°C for oral, axillary or tympanic route, or ≥ 38.0°C on rectal route.
  - Subjects with a minor illness (such as mild diarrhoea, mild upper respiratory infection) without fever were to be enrolled at the discretion of the investigator.
- Administration of immunoglobulins and/or any blood products during the period starting 3 months before the dose of study vaccine, or planned administration during the study period.
- Drug and/ or alcohol abuse within the last 5 years.

**Supplementary tables and figures**

**Supplementary Table 1.** **Percentage of participants with anti-HBs concentrations ≥6.2 mIU/mL, ≥10 mIU/mL, and ≥100 mIU/mL, anamnestic response, and anti-HBs GMCs, by age (ATP cohort for immunogenicity)**

| **Timepoint** | **Age, years** | **N** | **≥6.2 mIU/mL**  **(95% CI)** | **≥10 mIU/mL**  **(95% CI)** | **≥100 mIU/mL**  **(95% CI)** | **Anamnestic response, %**  **(95% CI)** | **Antibody GMC, mIU/mL**  **(95% CI)** |
| --- | --- | --- | --- | --- | --- | --- | --- |
| **Pre-challenge** | 40–50 | 59 | 91.5 (81.3–97.2) | 88.1 (77.1–95.1) | 61.0 (47.4–73.5) | Not applicable | 167.9 (96.7–291.4) |
|  | 51–60 | 42 | 97.6 (87.4–99.9) | 92.9 (80.5–98.5) | 61.9 (45.6–76.4) | Not applicable | 210.8 (108.3–410.4) |
| **7 days post-challenge** | 40–50 | 59 | 98.3 (90.9–100.0) | 98.3 (90.9–100.0) | 93.2 (83.5–98.1) | 88.1 (77.1–95.1) | 4882.2 (2576.3–9251.7) |
|  | 51–60 | 42 | 97.6 (87.4–99.9) | 95.2 (83.8–99.4) | 90.5 (77.4–97.3) | 78.6 (63.2–89.7) | 2740.6 (1208.0–6217.3) |
| **30 days post-challenge** | 40–50 | 59 | 100.0 (93.9–100.0) | 100.0 (93.9–100.0) | 98.3 (90.9–100.0) | 100.0 (93.9–100.0) | 44637.6 (26939.1–73963.8) |
|  | 51–60 | 42 | 100.0 (91.6–100.0) | 100.0 (91.6–100.0) | 97.6 (87.4–99.9) | 100.0 (91.6–100.0) | 55855.0 (30912.2–100923.9) |

Anti-HBs, antibodies against hepatitis B surface antigen; ATP, according-to-protocol; N, number of participants with available results; %, percentage of participants with concentration equal to or above specified value or percentage mounting an anamnestic response, defined as a post-challenge antibody concentration ≥10 mIU/mL for initially seronegative participants and a post-challenge antibody concentration at least four times the pre-challenge antibody concentration for initially seropositive participants (cut-off for seropositivity: 6.2 mIU/mL); GMC, geometric mean concentration; CI, confidence interval.

**Supplementary Table 2. Correlation between HBsAg-specific memory B cells or CD4^+^ T cells expressing a combination of at least two activation markers (among CD40L, IL2, IFNγ, and TNFα) and post-challenge anti-HBs concentrations or anamnestic response (ATP cohort for immunogenicity)**

| **CMI time point** | **Anti-HBs timepoint** | **N** | **Spearman-rank correlation coefficient (95% CI)** | |
| --- | --- | --- | --- | --- |
|  |  |  | **CMI and log10(anti-HBs concentration)** | **CMI and anamnestic response** |
| **Memory B cells** | | | | |
| **Pre** | **7 days post** | 66 | 0.34 (0.11–0.54) | 0.01 (-0.23–0.26) |
| **Pre** | **30 days post** | 66 | 0.26 (0.02–0.47) | -0.22 (-0.44–0.02) |
| **7 days post** | **7 days post** | 63 | 0.76 (0.63–0.85) | 0.41 (0.18–0.60) |
| **30 days post** | **30 days post** | 64 | 0.54 (0.34–0.70) | 0.13 (-0.12–0.36) |
| **CD4^+^ T cells** | | | | |
| **Pre** | **7 days post** | 70 | 0.14 (-0.10–0.36) | 0.01 (-0.23–0.24) |
| **Pre** | **30 days post** | 70 | 0.28 (0.04–0.48) | 0.11 (-0.13–0.33) |
| **7 days post** | **7 days post** | 63 | 0.23 (-0.02–0.45) | 0.09 (-0.16–0.33) |
| **30 days post** | **30 days post** | 65 | 0.40 (0.17–0.58) | 0.30 (0.06–0.51) |

HBsAg, hepatitis B surface antigen; CD40L, CD40 ligand; IL2, interleukin 2; IFNγ, interferon gamma; TNFα, tumor necrosis factor alpha; anti-HBs, antibodies against hepatitis B surface antigen; ATP, according-to-protocol; N, number of participants with available results; CI, confidence interval; CMI, cell-mediated immunity.

**Supplementary Table 3. Incidence of solicited symptoms reported within 4 days after the challenge dose (total vaccinated cohort)**

|  |  |  | **HBsAg vaccine group**  **N=103** |
| --- | --- | --- | --- |
| **Symptom** | **Type** | **n** | **%** (**95% CI**) |
| **Local (injection site)** |  |  |  |
| **Pain** | All | 40 | 38.8 (29.4–48.9) |
|  | Grade 3 | 0 | 0.0 (0.0–3.5) |
| **Redness** | All | 4 | 3.9 (1.1–9.6) |
|  | >50 mm | 0 | 0.0 (0.0–3.5) |
| **Swelling** | All | 3 | 2.9 (0.6–8.3) |
|  | >50 mm | 0 | 0.0 (0.0–3.5) |
| **General** |  |  |  |
| **Fatigue** | All | 27 | 26.2 (18.0–35.8) |
|  | Grade 3 | 0 | 0.0 (0.0–3.5) |
| **Gastrointestinal symptoms** | All | 10 | 9.7 (4.8–17.1) |
|  | Grade 3 | 0 | 0.0 (0.0–3.5) |
| **Headache** | All | 20 | 19.4 (12.3–28.4) |
|  | Grade 3 | 0 | 0.0 (0.0–3.5) |
| **Fever** | All | 0 | 0.0 (0.0–3.5) |
|  | >39.0°C | 0 | 0.0 (0.0–3.5) |

N, number of participants with the documented dose; n, number of participants reporting the symptom at least once; %, percentage of participants reporting the symptom at least once; CI, confidence interval; grade 3 defined as significant pain at rest or pain that prevented normal activity, headache, fatigue or gastrointestinal symptoms that prevented normal activity.

**Supplementary Figure 1. Fold-increases in anti-HBs concentrations from pre- to post-challenge in function of pre-challenge anti-HBs concentrations (ATP cohort for immunogenicity)**

Anti-HBs, antibodies against hepatitis B surface antigen; ATP, according-to-protocol. A and C show fold-increases from pre- to 7-days post-challenge, B and D from pre- to 30 days post-challenge. Blue circles and red squares in A and B are data points for participants who received a prior three- or four-dose HBsAg vaccination schedule, respectively. Blue circles and red squares in C and D are data points for participants in the 40–50 years and 51–60 years age groups, respectively.

**Supplementary Figure 2. Frequencies of HBsAg-specific IgG-producing memory B cells per million of IgG-producing memory B cells (A) and of HBsAg-specific CD4^+^ T cells expressing a combination of at least two immune markers (among CD40L, IL2, IFNγ, and TNFα) per million of CD4^+^ T cells (B), by age (ATP cohort for immunogenicity)**

HBsAg, hepatitis B surface antigen; IgG, immunoglobulin G; CD40L, CD40 ligand; IL2, interleukin 2; IFNγ, interferon gamma; TNFα, tumor necrosis factor alpha; ATP, according-to-protocol; N, number of participants with available results; Y, years of age; Q1–Q3, interquartile range. The x symbols in the boxes depict geometric mean frequencies.
